# Venous thrombotic events in psoriasis patients: a systematic review with meta-analysis

**DOI:** 10.1080/07853890.2021.1942974

**Published:** 2021-06-29

**Authors:** Tom Hillary, Jolien Clijmans, Séverine Vermeire, Jo Lambert, Marjan Garmyn, Maya Imbrechts, Thomas Vanassche

**Affiliations:** a Department of Dermatology, University Hospitals Leuven, Leuven, Belgium; b Katholieke Universiteit Leuven, Leuven, Belgium; c Department of Gastroenterology and Hepatology, University Hospitals Leuven and Translational Research in Gastrointestinal Disorders (TARGID) KU Leuven, Leuven, Belgium; d Department of Dermatology, Ghent University Hospital, Ghent, Belgium; e Department of Pharmaceutical and Pharmacological Sciences, Laboratory for Therapeutic and Diagnostic Antibodies, KU Leuven, Leuven, Belgium; f Department of Cardiovascular Diseases, University Hospitals Leuven, Leuven, Belgium

**Keywords:** Psoriasis, venous thromboembolism, comorbidities, deep venous thrombosis, pulmonary embolism, retinal vein occlusion

## Abstract

**Background:**

Psoriasis is a chronic inflammatory skin disease associated with numerous comorbidities. Psoriasis has been linked to an increased risk of metabolic syndrome and atherosclerotic arterial disease. Inflammatory conditions are known to increase the risk of venous thromboembolism (VTE), a frequent cause of morbidity and mortality. However, the relationship between psoriasis and VTE has received little attention and existing studies have shown conflicting results.

**Objectives:**

This systematic review aims to perform a meta-analysis on VTE in psoriasis patients.

**Methods:**

We conducted a systematic electronic search of the incidence of VTE (pulmonary embolism [PE], deep venous thrombosis [DVT] and/or retinal vein occlusion [RVO]) in psoriasis patients on PubMed, Web of Science, Embase and Cochrane (specifics: see Appendix 1 in Supporting information). Only English literature and full manuscripts were included; abstracts were excluded. Pooled risk ratio and 95% confidence interval were calculated using Review Manager.

**Results:**

Seven articles were included. Each study separately indicated a correlation between psoriasis and VTE after adjustment for several clinical parameters. The confounders included in the adjustment differed between studies, but all included adjustment for age, gender and comorbidities. A meta-analysis of the unadjusted data of the five studies that reported raw data on number of VTE events and patient follow-up (person-years) showed a pooled risk ratio for VTE and psoriasis of 1.29 (95% CI: 0.92–1.81). The statistical heterogeneity was high with *I*^2^ of 97%.

**Conclusions:**

Published data adjusted for key confounders demonstrate in general a significantly increased prevalence of VTE in psoriasis patients. Both psoriasis severity and number of confounders assessed seem to have an impact on this correlation. In this review, we pooled unadjusted data of the studies and we found a non-significant increased risk for VTE in psoriasis patients compared to healthy controls. This discrepancy suggests that psoriasis severity, age, gender or comorbidities may influence the risk of VTE in subgroups of the psoriasis population. Future research to identify subgroups at risk for VTE is warranted.

**Key messages**
- The included studies reported an increased risk of VTE, DVT, PE and RVO in psoriasis patients.
- A meta-analysis was performed on five studies that reported raw data and showed that the pooled risk ratio for VTE in psoriasis patients overall was increased, however not significantly, compared to healthy controls.
- Further research to pinpoint psoriasis subgroups at risk (e.g. severe psoriasis patients, younger age, associated comorbidities) of developing VTE is warranted.

## Introduction

Psoriasis is a common skin disease with a prevalence of up to 3% in the Western population [1]. It manifests as erythematosquamous plaques on the skin and can have major impact on quality of life. Histologically, it is characterized by downward elongation (comb-like) of the rete lists, parakeratosis and neutrophils in the epidermis (pustules of Kogoj and Munro micro-abscesses) [2]. Psoriasis is an auto-inflammatory disease with a strong genetic predisposition. Some of the genes involved encode for keratinocyte function although most seem to encode for components of inflammatory pathways (e.g. PSORS1), highlighting the disease’s inflammatory nature [3]. Psoriasis patients can present with a number of comorbidities, of which psoriatic arthritis (prevalence of up to 30%) is most known and can lead to joint deformity and major disability if left untreated [4]. In addition, psoriasis patients have a disease severity-dependent risk of associated conditions such as dyslipidaemia, visceral obesity and diabetes. Furthermore, the relative risk of myocardial infarction (stroke) in young males with severe psoriasis is increased up to 3.1 compared to healthy peers [5,6].

Venous thromboembolism (VTE) in the general population is a frequent and possibly fatal condition, often provoked by classical risk factors [7] (Table 1). An estimated incidence among people of European ancestry of 104–183 cases per 100,000 person-years [8]. It has an annual mortality rate of 6% for deep vein thrombosis (DVT) and 12% for pulmonary embolism (PE) [9]. According to Grosse et al. [10], treatment of an acute VTE on average is associated with incremental direct medical costs of $12,000–15,000 (2014 US dollars) among first-year survivors. This correlates with a burden on the annual health care budget in the USA of $7–10 billion each year.

**Table 1.** Potential risk factors for definite deep vein thrombosis or pulmonary embolism (adapted from Petitpain et al.).

Institutionalization with or without recent surgery
Trauma
Malignant neoplasm with or without chemotherapy
Prior central venous catheter or transvenous pacemaker
Prior superficial vein thrombosis
Neurologic disease with extremity paresis
Varicose veins
Congestive heart failure
Serious liver disease
Age
Sex
Smoking
Corticosteroid use
Oral use of contraceptives
Family history of VTE

The three underlying factors that are thought to contribute to VTE are hypercoagulability, endothelial injury and venous stasis, also known as the triad of Virchow [11]. Both hypercoagulability and endothelial injury are observed in inflammatory diseases and thought to be caused by inflammation-induced upregulation of thrombogenic factors and increased attraction of circulating monocytes, respectively. Inflammation-induced upregulation of thrombogenic factors results in platelet aggregation and clot formation, while the increased attraction of monocytes leads to increased infiltration of vascular walls, resulting in vascular damage [12]. In addition to the aforementioned factors, neutrophils may play a role in VTE: due to their ability to adhere to both platelets and endothelial surfaces, activated neutrophils are suggested as possible mechanisms linking inflammation and coagulation, thereby contributing to a prothrombotic state [13].

With this in mind, it is not surprising that an increased incidence of VTE is observed in patients with inflammatory diseases. Reported incidence rates (per 1000 years) of VTE are higher in patients with lupus erythematosus (LE) (both cutaneous and systemic): 1.20, 3.06 and 5.24 for the reference population, Cutaneous LE and Systemic LE, respectively. Also, patients with inflammatory bowel disease (IBD) are reported to have a 1.5- to 3-fold increase in the risk of VTE [14–16].

There is awareness among dermatologists that psoriasis patients are at increased risk of atherosclerosis and myocardial infarction among others, which are complications that take place in the arterial compartment of the blood vessels. Physicians, in general, recognize the importance of these comorbidities and guidelines encourage to screen for these associated conditions. In contrast, what happens in the venous compartment of the blood system is less extensively studied and no recent synthesis of these data is available. Iskandar et al. reported the prevalence of DVT and PE in a British psoriasis cohort: 0.6% and 0.4%, respectively, with no difference between a cohort treated with biologics and a cohort treated with traditional systemic treatments [17]. In 2014, Ungprasert et al. published a meta-analysis on the incidence of VTE in psoriasis and reported a pooled risk ratio of VTE in subjects with psoriasis of 1.46 (95% CI: 1.29–1.66) [18]. However, the statistical heterogeneity was high with an *I*^2^ of 86% and statistics was performed on adjusted data. Since then, new data on this topic were published.

Furthermore, for JAK inhibitors, a recently developed therapeutic approach for inflammatory diseases, safety signals involving increased risk of VTE in clinical trials were observed, already resulting in several boxed warnings from the US Food and Drug Administration [19,20]. Stratification of patients with increased risk of VTE is warranted, in order to make optimal therapeutic decisions and avoid potentially thrombogenic anti-inflammatory therapies in patients at risk.

Therefore, we reviewed the literature on the occurrence of VTE in psoriasis with emphasis both on deep venous thrombosis (DVT), PE and retinal vein occlusion (RVO) and performed a meta-analysis.

## Materials and methods

### Search strategy and data source

Prior to an online search in PubMed, Embase, Web of Science and Cochrane was conducted to identify articles reporting on psoriasis and VTE, the study was registered on PROSPERO (registration number: CRD42019125145). Specifics of the search can be found in Appendix 1 in Supporting information. Inclusion of papers was performed by two independent reviewers (TH and JC) based on Title and Abstract using the following inclusion criteria: (i) observational studies published as original studies, (ii) data reported on one or more of the following outcomes: risk of VTE, DVT, PE or RVO in patients with psoriasis.

Original data from systematic reviews, cohort studies, case-control studies and cross-sectional studies were included. Exclusion criteria were as follows: (i) language not in English or Dutch, (ii) case reports and case series or (iii) other study design than observational studies with a publication date before 2000.

Disagreement between the reviewers was solved by re-reading and discussion. In addition to the electronic search, a manual search of reference lists of included studies, reviews and meta-analyses was performed for relevant articles.

Quality assessment was performed for each included article according to PRISMA guidelines using the National Heart, Lung and Blood Institute Study Quality Assessment tools. We applied the quality assessment tool for observational cohort and cross-sectional studies on each study (Appendix 2 in Supporting information).

### Data extraction

Data extraction was done by one reviewer (JC) using a data collection template (Excel spreadsheet MS Office 2016).

A standardized data collection form was used to extract the following information: (1) last name of the first author; (2) publication year; (3) study design; (4) country where the study was conducted; (5) study size including numbers of patients with psoriasis and number of controls; (6) homogeneity of study population including percentage of women and mean age; (7) period of follow-up; (8) average range of follow-up; (9) method used for the verification of psoriasis and VTE, PE, DVT or RVO; (10) outcome measures including VTE, DVT, PE and RVO; (11) confounders assessed in adjusted statistical models. An overview of the confounders can be found in Table 2.

**Table 2.** List of the seven studies with the confounders that were included in the adjusted analysis.

Author	Confounders assessed
Chung WS, Lin CL	Sex, age, comorbidities (hypertension, diabetes, CVA, heart failure, all cancer, pregnancy, atrial fibrillation, lower leg fracture of surgery, asthma, CAD, COPD, RA, obesity)
Ahlehoff O, Gislason GH, Lindhardsen J, et al.	Age, malignancy, surgery, rheumatological disease
Lutsey PL, Prizment AE, Folsom AR	Age, education, smoking status, BMI, DM, HRT use
Ogdie A, McGill NK, Shin DB, et al.	joint replacement surgery, malignancy, heart of respiratory failure, pregnancy, hormone therapy, smoking, alcohol intake, BMI, hospitalization during the baseline period, year of cohort entry, socioeconomic status, urban versus rural living environment, Charlson comorbidity score, chronic kidney disease, myocardial infarction, atrial fibrillation, diabetes, hypertension, COPD, liver disease, disability status, start year in the cohort and interaction between disease status and DMARD use
Ramagopalan SV, Wotton CJ, Handel AE, et al.	Age, sex, region of residence
Zöller B, Li X, Sundquist J, et al.	Sex, age, hospitalization, COPD, obesity, alcohol use, CAD, stroke, hypertension, varicose vein, sepsis, CHF, PVD
Yen YC, Weng SF, Lai FJ, et al.	Sex, age, comorbidities (DM, HTN, CHD, hyperlipidaemia, obesity and COPD)

### Statistical analysis

Review Manager software (version 5.3) from the Cochrane collaboration was used for the data analysis. A number of events (VTE) and years of follow-up (person-years) were abstracted from the studies (five) and a Mantel Haenszel analysis was performed [21]. A random-effect model was used. Statistical heterogeneity was high (*I*^2^ = 97%).

## Results

### Overview of articles

**Figure 1.**
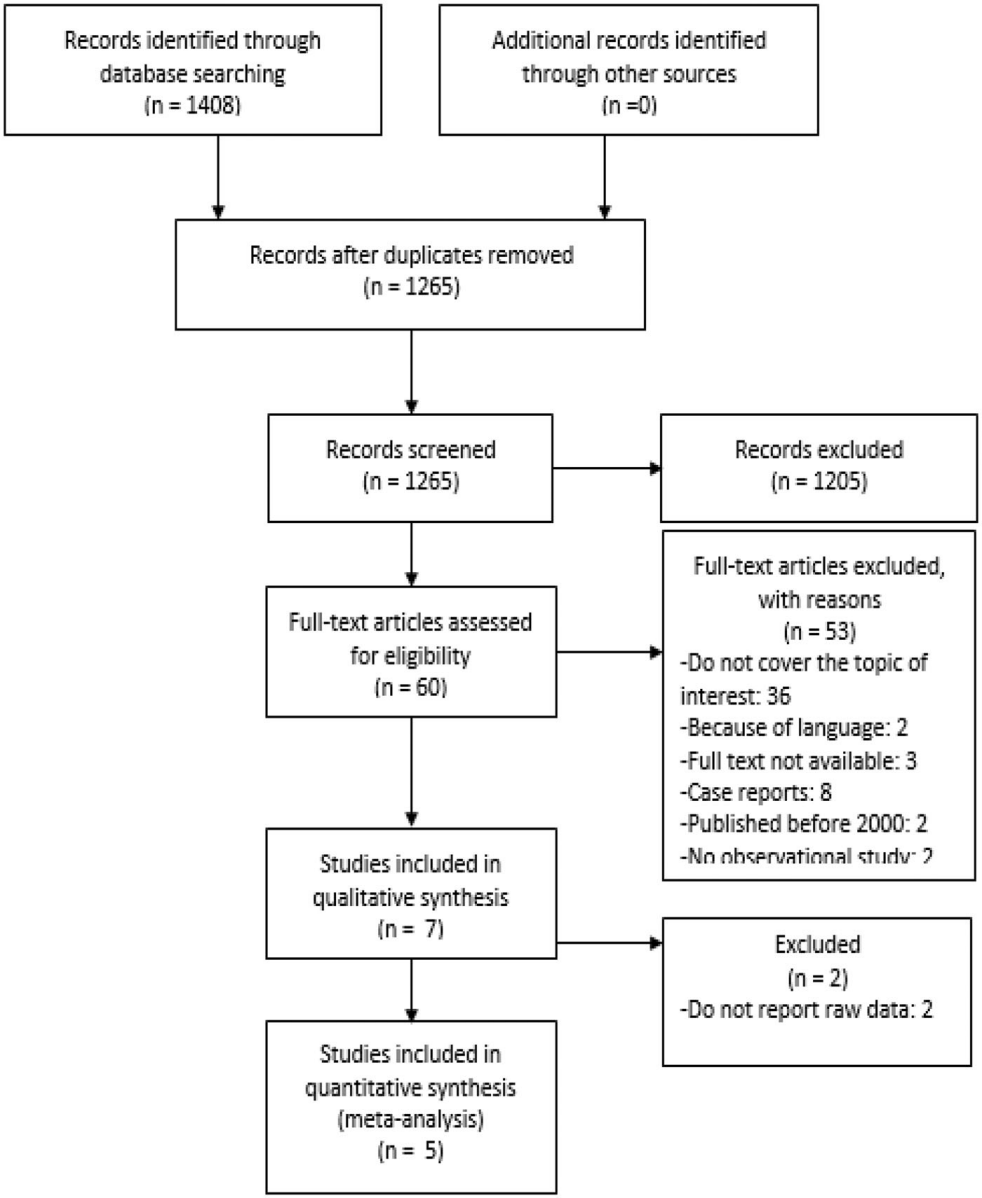
Flowchart of article selection. Adapted from Moher et al. [28].

A total of 1408 articles were screened. After the removal of duplicates, 1265 original papers remained. Seven articles were included based on agreement of the two reviewers after reading title and abstract [12,22–27]. A flowchart of article selection is presented in Figure 1 [28]. Some articles report on VTE, other articles make distinction between DVT, PE and RVO. An overview of the included articles and reported data is provided in Appendix 3 in Supporting information.

### Graphic display of reported data in seven studies

**Figure 2.**
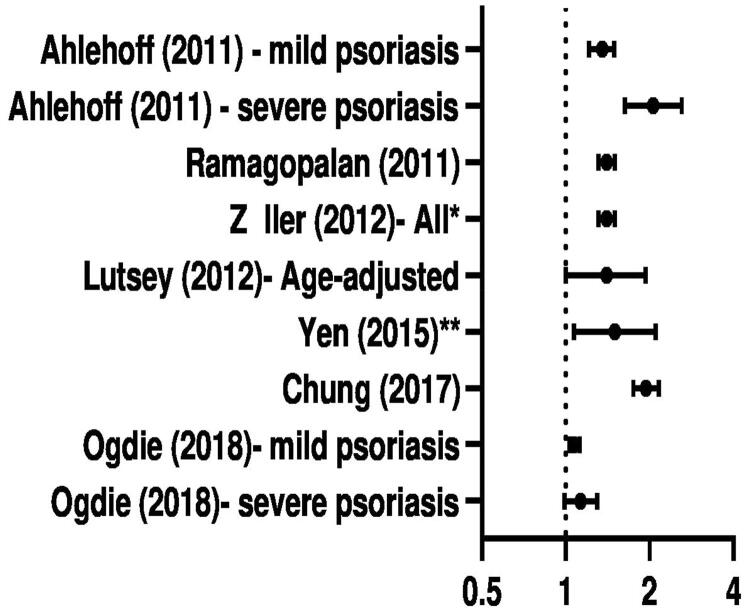
Overview of reported data on psoriasis and VTE using the adjusted data. Details of adjustment varied between studies (as shown in Table 1). * reports on PE; ** reports on RVO (reported data: Ahlehoff: Rate Ratio; Ramagopalan: Rate Ratio; Zöller: SIR; Lutsey: HR; Yen: aHR; Chung: Incidence Rate Ratio; Ogdie: aHR).

The reported relative risk for VTE in psoriasis patients for the seven individual studies is visualized in Figure 2. In most studies, a significantly increased incidence of VTE in psoriasis patients is observed. Subgroup analysis of severe psoriasis was performed in two studies [12,26], both studies observed that more severe psoriasis had higher incidence of VTE, compared to mild psoriasis. Ahlehoff et al. reported incidence rates per 1000 person-years of 1.29, 1.92 and 3.20 for healthy controls, mild and severe psoriasis, respectively. Ogdie et al. reported incidence per 10,000 person-years of 38.67, 36.25 and 44.80 for healthy controls, mild and severe psoriasis, respectively.

**Figure 3.**
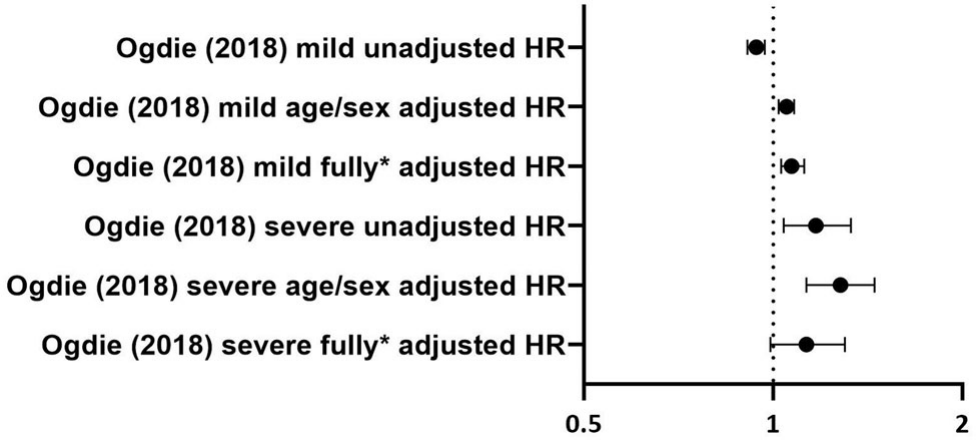
Overview of the reported hazard ratios in the study of Ogdie et al. on DVT. Dots show HR (hazard ratio); lines represent 95% confidence interval; * shows adjustment for sex, age, hypertension, history of cancer, joint replacement in the baseline period, hospitalization in the baseline period, chronic obstructive pulmonary disease, liver disease, oral glucocorticoids, NSAIDs, smoking and drinking in the baseline period.

**Figure 4.**
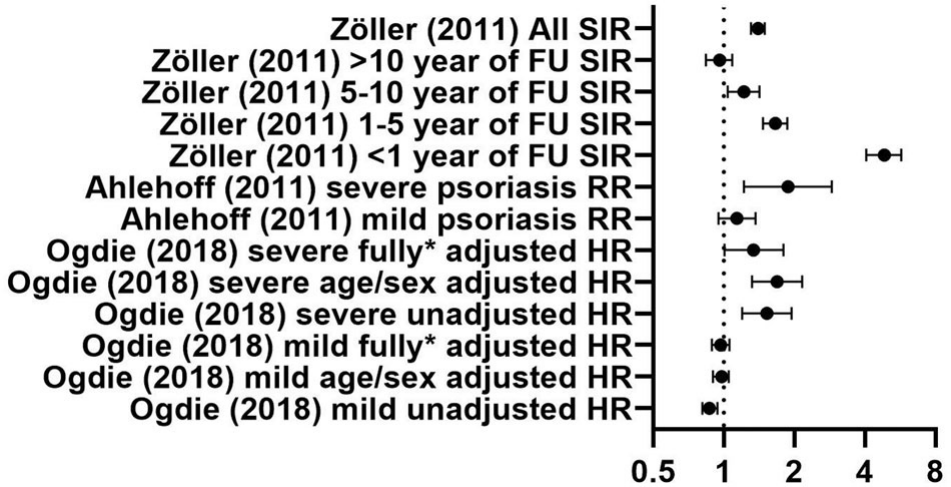
Overview of reported ratios of the included studies on PE. Dots show SIR (standardized incidence ratio), RR (Rate Ratio) , HR (hazard ratio); lines represent 95% confidence interval; * shows adjustment for sex, age, hypertension, history of cancer, joint replacement in the baseline period, hospitalization in the baseline period, chronic obstructive pulmonary disease, liver disease, oral glucocorticoids, NSAIDs, smoking and drinking in the baseline period.

Figures 3 and 4 give a graphic overview of reported data on DVT and PE, respectively. Ogdie et al. reported on the hazard ratio (HR) of DVT in both mild and severe psoriasis patients. After adjustment for age/sex, a significant increase in DVT was seen in both mild and severe psoriasis.

For PE, the reported standardized incidence ratios (SIR), relative risks and HR show a clear trend towards increased risk in psoriasis patients. In the subgroup of severe psoriasis patients, this increase was significant in all studies.

### Five studies eligible for meta-analysis

**Figure 5.**
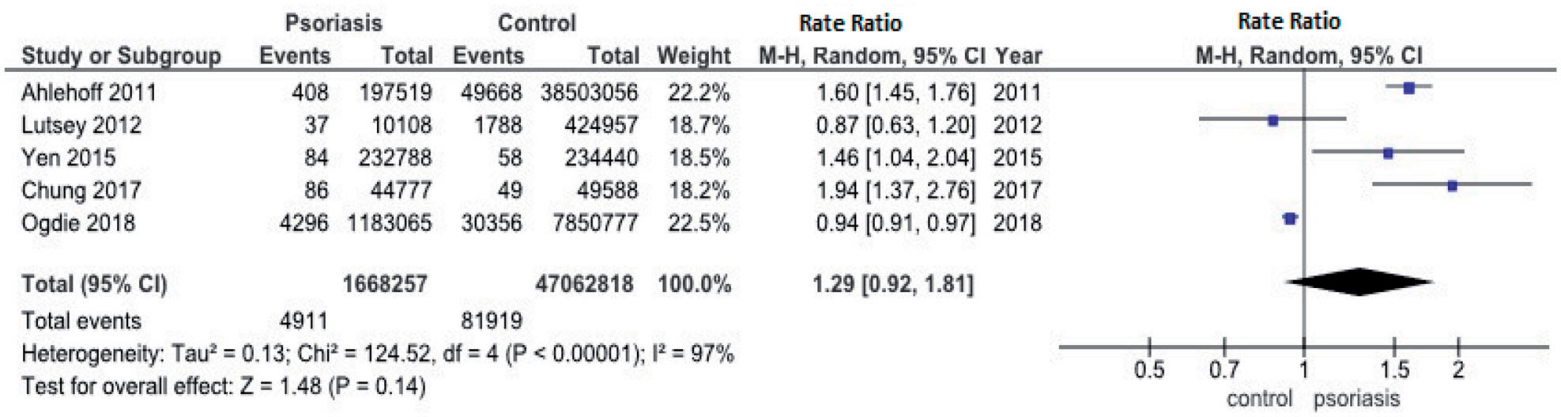
Forest plot of the studies included for meta-analysis. On the left side, data for statistical analysis are presented.

Five studies reported raw data on number of events and time of follow-up (person-years). A meta-analysis on these unadjusted data was performed (Figure 5). The pooled risk ratio for VTE in psoriasis patients was 1.29 (95% CI: 0.92–1.81). The statistical heterogeneity was high with *I*^2^ of 97%.

## Discussion

In this systematic review and meta-analysis, we studied the problem of VTE in patients with psoriasis. The reported adjusted data of individual studies suggested an association between psoriasis and VTE although data were somewhat conflicting.

Our meta-analysis, performed on the raw data of 1,668,257 psoriasis person-years and 47,062,818 control person-years shows a non-significant increased risk for venous thromboembolic events with a pooled risk ratio of 1.29 (95% CI: 0.92, 1.81). All studies were of (relatively) high quality. Publications were not commercially funded, and both significant and non-significant results were published: we therefore believe the included reports are not prone to bias.

In contrast, the reported (adjusted) data for VTE, PE, DVT and RVO, from the individual studies show a clear trend to a significant increased risk in psoriasis patients, especially in groups with severe psoriasis.

The discrepancy between the adjusted and unadjusted data suggests that the factors included as confounders influence the risk of VTE in patients with psoriasis. The individual studies showed important differences between the psoriasis group and the selected group of healthy controls, in addition to the presence or absence of psoriasis. Although the individual studies used different variables (Table 2), all included age, gender and comorbidities. Therefore, these factors should be considered when evaluating the risk of VTE.

Previously, Ungprasert et al. reported in their review a statistically significant increased VTE risk among patients with psoriasis after processing adjusted data. The addition of more recent data seems to influence the results in our meta-analysis: it is important to emphasize that our statistical analysis was performed on raw data only (patient-years and numbers of event). Furthermore, the cohort of Ogdie et al. contributes significantly to the overall patient-years. We believe their definition of severe psoriasis (treatment with phototherapy or Disease Modifying Anti-Rheumatic Drugs (DMARDs)) can be a source of bias, since a significant proportion of patients – who may classify as having severe disease – cannot receive DMARDs due to comorbidities or refuses systemic therapy. Hence, there may have been an underrepresentation of patients with severe disease. In addition, inclusion in the cohort was based on diagnostic codes in a general practitioner’s research database and therefore may be prone to misdiagnosis. Overall, these factors are likely to contribute to the high heterogeneity of the analysis.

An important limitation of the included studies is the retrospective nature: patient data were included from patients registries and these registries are not specifically designed to evaluate the severity of psoriasis or incidence of VTE in psoriasis patients. Moreover, the reported databases differ geographically and ethnically.

Despite the overall reassuring results of the pooled risk ratio, some psoriasis patients are at increased risk for VTE. Some efforts for stratification of patients have been undertaken previously. Ahlehoff et al. reported a significantly higher risk for VTE in the younger psoriasis population (<50 years old) compared to the older group (>50 years old). Also, Gelfand et al. reported that young psoriasis patients are significantly more at risk to suffer from myocardial infarction than older patients [5]. Possibly young age may be a surrogate marker for more severe inflammation and disease. Other groups also report an increased risk for VTE in more severe psoriasis groups. This finding is in line with other reported comorbidities of psoriasis [29,30]. However, this signal is not consistent over all studies possibly because definitions for disease severity have been very different across studies. Importantly, no objective scoring systems (psoriasis area severity index [PASI], body surface area [BSA]) were applied in the assessment of severity.

Ramagopalan et al. looked into the risk of VTE (defined as DVT or PE) in people admitted to hospital – a known risk factor for VTE – with a range of immune-mediated diseases [22]. Interestingly, the authors studied the occurrence of VTE in time intervals after admission. By subdividing the results into a period of 0–90 days and a period of 91+ days, they demonstrated that psoriasis was associated with increased VTE risk at both short- and long term. Finally, Zöller et al. investigated all individuals in Sweden that were hospitalized for PE and had a primary or secondary diagnosis of an autoimmune disorder between 1964 and 2008 [24]. A significant increased risk was reported in the psoriasis cohort compared with controls, with a strong correlation to disease duration: the shorter the time of diagnosis, the higher the SIR. This finding could be explained by the fact that it often takes years before psoriasis patients receive proper care and supports psoriasis severity as a risk factor for VTE [31].

Current American guidelines take into account the psoriatic march (systemic inflammation leading to consecutively insulin resistance, endothelial dysfunction, atherosclerosis and ultimately myocardial infarction or stroke) but do not mention psoriasis as a possible risk factor for VTE, nor do they advise on thrombosis prophylaxis in psoriasis patients [6,32]. Among the European standards, only the guideline from the National Institute for Health and Care Excellence (United Kingdom) mentions VTE [33]. In conclusion, although individual studies suggest a correlation between psoriasis and VTE, our meta-analysis of unadjusted data could not confirm a significant increased risk for VTE in all psoriasis patients. The discrepancy between adjusted and unadjusted data likely reflects differences in VTE risk factors between psoriasis patients and the groups selected as healthy controls.

In conclusion, future research should focus on identifying subgroups among psoriasis patients at risk of developing VTE. The discrepancy between adjusted data and unadjusted data also implicates a significant role of some – but not all – key confounders. Furthermore, patients with increased inflammatory burden due to associated diseases (psoriatic arthritis, IBD) or more severe disease (defined by objective disease scores) should be targeted in our opinion. Future guidelines should consequently incorporate these findings, and the addition of any classical risk factor (Table 1) of VTE in psoriasis patients with a high-risk profile of VTE should trigger initiation of preventive measurement (e.g. stockings) or prophylactic therapy.

## Data Availability

The data that support the findings of this study are available on request from the corresponding author (T.H.).
